# Discriminant equation using mucosally expressed cytokines and transcription factor for making definite diagnosis of inflammatory bowel disease unclassified

**DOI:** 10.1186/s12876-021-01656-1

**Published:** 2021-02-16

**Authors:** Hiroaki Okuno, Haruei Ogino, Eikichi Ihara, Kei Nishioka, Yoshimasa Tanaka, Takatoshi Chinen, Motoyuki Kohjima, Takamasa Oono, Masatake Tanaka, Takeshi Goya, Nao Fujimori, Yoichiro Iboshi, Takuji Gotoda, Yoshihiro Ogawa

**Affiliations:** 1 Department of Medicine and Bioregulatory Science, Graduate School of Medical Sciences, Kyushu University, 3-1-1 Maidashi, Higashi-ku, Fukuoka, 812-8582 Japan; 2 Division of Gastroenterology and Hepatology, Department of Medicine, Nihon University School of Medicine, Tokyo, Japan; 3 Department of Gastroenterology and Metabolism, Graduate School of Medical Sciences, Kyushu University, Fukuoka, Japan; 4 Department of Gastroenterology, Clinical Research Institute, National Hospital Organization, Kyushu Medical Center, Fukuoka, Japan

**Keywords:** Ulcerative colitis, Crohn’s disease, Inflammatory mediator

## Abstract

**Background:**

The pathological conditions of UC and CD involved in inflammatory bowel disease-unclassified (IBD-U), UC with primary sclerosing cholangitis (PSC-UC), and UC with autoimmune pancreatitis type 2 (AIP-UC) remain unclear. Therefore, it is difficult to decide the appropriate treatments for these subtypes of UC. Our aim was to examine whether the discriminant equation using the mucosally expressed mediators designed as our previous study for IBD, could characterize IBD-U, PSC-UC, or AIP-UC.

**Methods:**

A total of 56 patients including UC (n = 24), CD (n = 15), IBD-U (n = 10), PSC-UC (n = 4), and AIP-UC (n = 3), along with 9 control patients were enrolled in this study. Mucosally expressed inflammatory mediators related to Th1, Th2, Th17, and Treg were measured using quantitative PCR in endoscopic biopsies from the inflamed intestines of the patients. The IBD-U, PSC-UC or AIP-UC were characterized using discriminant analysis and principle component analysis.

**Results:**

Through discriminant analyses, combinations of 3 to 7 inflammatory mediators were used to discriminate between UC and CD. Moreover, the identified 3 markers could diagnose patients with IBD-U as UC or CD with high accuracy. The distribution graph of inflammatory mediators using the principal component analysis revealed that PSC-UC and AIP-UC exhibited CD-like and UC-like features, respectively.

**Conclusions:**

The discriminant equation using mucosally expressed mediators of IL-13, IL-21 and T-bet can be used as a universal diagnostic tool not only for IBD-U but also to assess pathological conditions in PSC-UC and AIP-UC.

## Background

Inflammatory bowel diseases (IBD) have been classified as either ulcerative colitis (UC) or Crohn’s disease (CD), which are diagnosed based on a comprehensive assessment of the clinical course, endoscopy, and pathological findings [1]. However, UC and CD are heterogeneous diseases, including a variety of complex phenotypes. Therefore, approximately 2–9% of the patients with IBD do not meet the diagnostic criteria of UC or CD [2, 3]. These cases are classified as inflammatory bowel disease-unclassified (IBD-U) [4].

Although the underlying pathogenesis of IBD-U remains unclear, it could be considered as an early stage of UC or CD. It is difficult to decide which treatment of UC or CD is appropriate for patients with IBD-U. Indeed, there are significant differences in the therapeutic agents including applicable biologicals between UC and CD. Owing to the failure of established diagnosis and treatment of IBD-U, the prognosis of patients with IBD-U is worse than those with the conventional IBD, resulting in poor surgery outcomes and a higher risk of chronic pouchitis [5–7]. Thus, it is important to make a differential diagnosis of IBD-U, if applicable, to improve clinical outcomes and quality of life of the patients with IBD-U.

Furthermore, a special case of IBD that reflects the heterogeneity of pathogenesis is not only IBD-U but also UC, along with primary sclerosing cholangitis (PSC-UC) and autoimmune pancreatitis (AIP-UC). Especially, PSC-UC is reported to have characteristics different from those of conventional UC with regard to both clinical characteristics and immune reaction [8, 9]. In addition to IBD-U, it is important to evaluate the characteristics of PSC and AIP-UC.

We have previously reported that mucosally expressed inflammatory mediators can be distinguished between UC and CD [10]. On the basis of the difference in the expression of CD4^+^ T cell subsets in the intestinal mucosa between UC and CD [11], we created the discriminant equation to distinguish between them using the expression levels of inflammatory mediators from the inflammatory mucosa. Although this discriminant analysis using mucosally expressed inflammatory mediators is expected to be useful for diagnosis, monitoring, and defining the distinctive characteristics of IBD, it needs to be improved further. In particular, it is not clear what portion of the mucosa from the rectum to terminal ileum should be selected for the analysis as it is not known whether the expressions of inflammatory mediators were different between the colorectum and ileum in one of the patients.

The aim of this study was to examine the discriminant equation, using mucosally expressed mediators that we designed in accordance with our previous study for IBD, that could determine IBD-U, PSC-UC or AIP-UC.

## Methods

### Patients

**Fig. 1:**
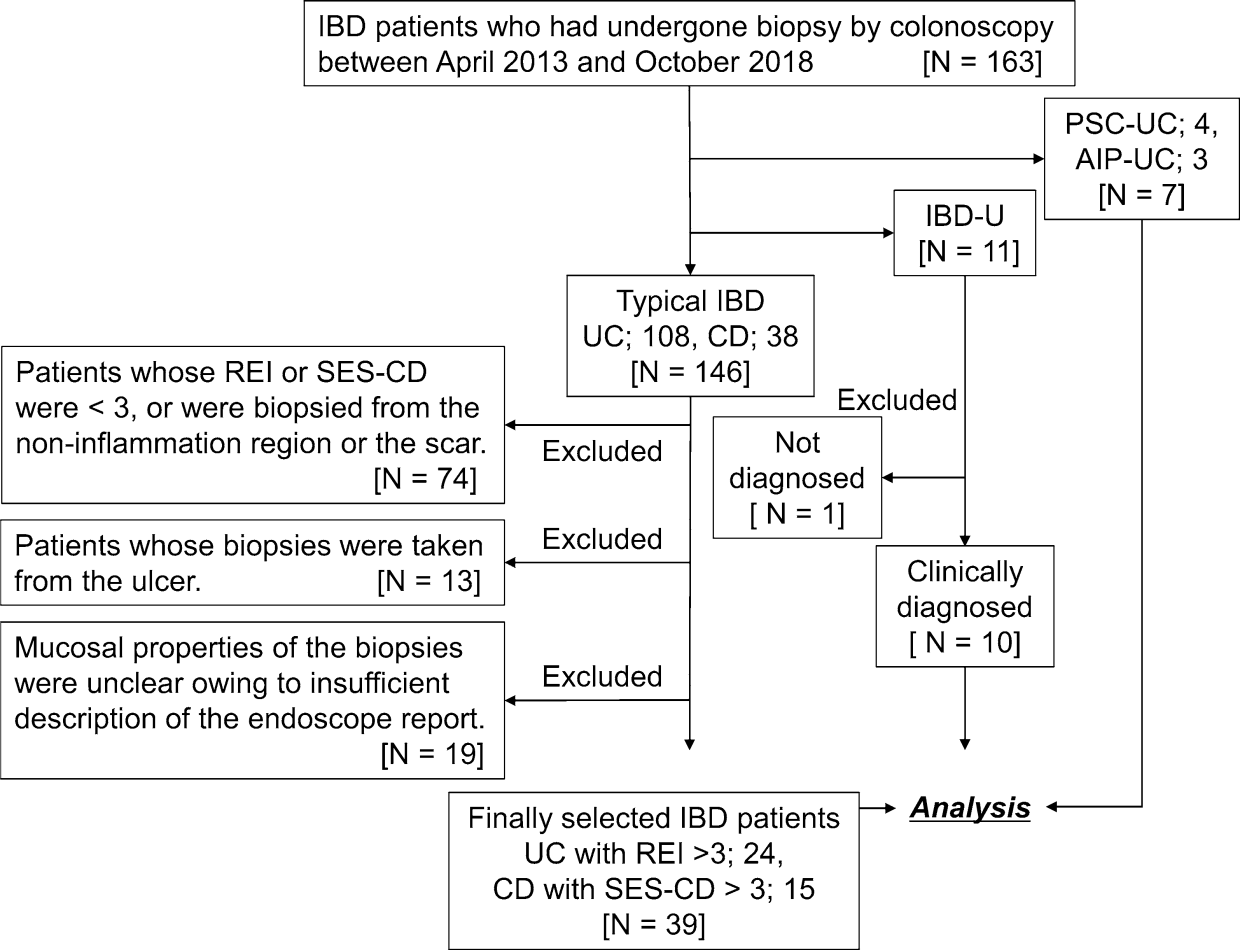
Patients flow-chart. *IBD* inflammatory bowel disease, *UC* ulcerative colitis, *CD* Crohn’s disease, *REI* Rachmilewitz endoscopic index, *SES-CD* simple endoscopic score for Crohn's disease

Between April 2013 and October 2018, a total of 163 patients with IBD who had undergone colonoscopy at Kyushu University Hospital and three other referral centers were primarily enrolled. Patients were divided into four groups at the first consultation: 146 patients with IBD (108 with UC, 38 with CD), 11 patients with IBD-U, 4 patients with PSC-UC, and 3 patients with AIP-UC. We excluded 74 patients with IBD who were considered to be in an inactive state, whose Rachmilewitz endoscopic index (REI) was 3 or less, whose simple endoscopic score for CD (SES-CD) was 3 or less, or in whom biopsy was performed from a noninflamed region. In addition, 13 patients, from whom an ulcer biopsy sample was taken, were excluded since the biopsy samples had necrotic tissue. One patient with IBD-U who has not been clinically diagnosed during this study was also excluded. Furthermore, 19 patients with IBD were excluded because their endoscopic reports contained an insufficient description of the properties of the biopsy site. Finally, 24 patients with UC, 15 patients with CD, 10 patients with IBD-U, 4 patients with PSC-UC, and 3 patients with AIP-UC were included (Fig. 1).

### Classifications and definitions

After excluding infections, ischemia, and other specific forms of colitis, the diagnoses of UC and CD were made base on conventionally clinical, laboratory, imaging, and endoscopic parameters, including histopathology [1, 12, 13]. All patients underwent esophagogastroduodenoscopy and total colonoscopy at the first visit. Computed tomography and/or magnetic resonance imaging were also performed consecutively on patients as soon as possible after the first visit. Additionally, in patients with suspected CD or IBD-U, double-balloon enteroscopy or barium examination of the small intestine was performed. Patients who did not meet all the criteria but had the features of IBD that cannot be classified as UC or CD were designated as IBD-U [1, 3]. According to the Montreal classification, IBD-U is suggested for patients in whom there is clinical and endoscopic evidence for IBD affecting the colon, without small bowel involvement [4]. However, recently, there have been a number of patients with UC who had small bowel lesions like backwash ileitis [14]. Therefore, in this study, even if there was a lesion in the terminal ileum, it was defined as IBD-U when it did not meet the diagnostic criteria for UC or CD. Patients with IBD-U who met the criteria for UC or CD within a long-term observation of their clinical course, endoscopic parameters, pathology, and surgery were defined as candidates for this study. PSC and AIP were diagnosed following the relevant criteria [15, 16].

We calculated the REI, which is evaluated using several endoscopic parameters, namely, vascular pattern, granulation, mucosal vulnerability, fibrin, exudate, erosion, and ulcers, for patients with UC [17], and we also evaluated SES-CD, which is based on the ulcer size, ulcerated surface, affected surface, and presence of luminal narrowing, for patients with CD [18]. In addition, we collected information regarding the age, sex, disease duration, disease type, and treatment received of individual patients.

### Sample collection

Biopsy samples were obtained by colonoscopy from inflamed areas of the colorectum and/or terminal ileum in patients with UC or CD. Inflamed areas were defined as areas with the most severe inflammation, with typically edema, friable granular mucosae, marked erythema, disappearance of vascular patterns, erosions, spontaneous bleeding, or the margin of ulcer formation [10]. Biopsy samples were collected from two different locations: exclusively from the colorectum in 6 patients with UC and from the colorectum and/or terminal ileum in 4 patients with CD. The samples were analyzed to confirm whether there was a difference in the pattern of inflammatory mediator expression at different locations. Control patients were defined as those scheduled to undergo colonoscopy for the treatment of colon polyps, and their biopsy samples were taken from normal mucosa of the colorectum. All samples obtained from the patients were immediately soaked in RNAlater (Ambion, Austin, TX, USA) after biopsy and stored at − 30℃ after overnight incubation at 4℃.

### Extraction of RNA and quantitative real-time PCR

Gene expression analysis was performed in the same methods as we previously reported [19]. Total RNA was extracted from the biopsy tissues using TRIzol reagents (Invitrogen, Carlsbad, CA, USA), and RNA concentrations were measured at 260/280 nm using UV spectrophotometry. RNA (2 mg) from each sample was reverse transcribed using the QuantiTect Reverse Transcription Kit (Qiagen, Valencia, CA, USA). Synthesized complementary DNA equivalent to 20 ng of RNA was mixed with TaqMan Universal PCR Master Mix, No AmpErase UNG (Applied Biosystems, Foster City, CA, USA), in wells up to a volume of 20 µL. Quantitative real-time PCR was performed on a CFX96 Real-Time PCR Detection System (Bio-Rad, Hercules, CA, USA) in a 96-well format. The expression levels of genes encoding the following inflammatory mediators were determined using FAM-labeled TaqMan Gene Expression Assay reagents (Applied Biosystems); TNF-α, IFN-γ, IL-12 p35, IL-12 p40, T-bet, IL-5, IL-13, IL-33, GATA3, IL-17A, IL-17F, IL-21, IL-22, IL-23 p19, IL-6, RORC, TGF-β, and FoxP3. Additional file 1: Table S1 shows the product numbers of the kits used to evaluate the expression of all genes.

The reaction conditions used for the quantitative real-time PCR were as follows: 95℃ for 10 min followed by 40 cycles of 95℃ for 15 s and 60℃ for 60 s. All assays were performed in duplicate. 18S ribosomal RNA, as detected using gene expression assay Hs99999901_s1 (Applied Biosystems), was used to normalize the expression levels of the target genes. Standard curves were prepared for each assay using 10 different standard preparations and were considered sufficiently stable.

### Data and statistical analysis

First, ΔCq values were acquired by subtracting the threshold cycle (Cq) of the 18S ribosomal RNA from those of the target genes. Next, we divided 1 by the ΔCq values to calculate the reciprocal ΔCq values, which we then used for subsequent statistical analyses. Reciprocal ΔCq values were defined as 0 when the target genes were not detected in 40 cycles of real-time PCR.

Analysis of the distribution of inflammatory mediator expression in different location of the lesions was performed using principal component analysis (PCA). Principal component analysis (PCA) can help simplify large datasets by creating new uncorrelated variables that continuously maximize the variance. It can also improve interpretability without the loss of valuable information. In addition, drawing 3D scatterplots of principal components (PCs) helped visualize the parameter distribution in UC and CD.

The discriminant ability between UC and CD was analyzed using discriminant equations with 7 parameters (TNF-α, IL-12 p40, T-bet, IL-13, GATA3, IL-21, and RORC), with 5 parameters (IFN-γ, T-bet, IL-12 p35, GATA3, and IL-21), and with 3 parameters (T-bet, IL-13, and IL-21) that were created using linear discriminant analysis (LDA) in our previous research. In each discriminant equation, an appropriate coefficient was determined using LDA. As indices of discrimination, we calculated the areas under the receiver operating characteristic curves (AUCs) and 95% confidence intervals (95% CIs). After establishing updated discriminant equations, we applied the reciprocal ΔCq values of IBD-U to each discriminant equation to confirm the prediction accuracy. Finally, we analyzed the distribution of inflammatory mediators in PSC-UC and AIP-UC by performing a PCA.

All statistical analyses were performed using JMP Pro 14.0.0 (SAS Institute, Inc., Cary, NC, USA). Statistical significance for differences in the target gene expression ratios was evaluated using the Kruskal–Wallis test and Dunn’s test, and *P* values < 0.05 were considered statistically significant.

## Results

### Patient characteristics

Table 1 shows the baseline clinical characteristics of the patients. In total, 65 patients were enrolled: 24 patients with UC, 15 patients with CD, 10 patients with IBD-U, 4 patients with PSC-UC, 3 patients with AIP-UC, and 9 patients included in the control group. Patients with UC, CD, IBD-U, PSC-UC, and AIP-UC were younger than those in the control group. Biopsy specimens were taken from the colorectum from all patients with UC, whereas specimens were taken from the terminal ileum in 5 out of the 15 patients with CD. In addition, 11 out of the 24 (45.8%) patients with UC showed pancolitis, whereas no patients with PSC-UC or AIP-UC had pancolitis. Ileocolitis was observed in 4 out of the 10 (40%) patients with IBD-U. Additional file 1: Table S2 shows the clinical course of IBD-U patients. Finally, three patients with IBD-U were diagnosed with UC and seven with CD. One biopsy sample from each patient was used for subsequent analyses. No significant endoscopic complications were observed during the study period.

**Table 1:** Clinical characteristics of patients

	UC	CD	IBD-U	PSC-UC	AIP-UC	Control
N	24	15	10	4	3	9
*Sex*						
Male/female	11/13	10/5	7/3	4/0	2/1	8/1
Age^†^	41.0 (26.7–55.3)	33.0 (27.0–39.0)	36.5 (30.4–42.6)	33.5 (24.8–42.2)	49.0 (35.0–63.0)	68.0 (62.3–70.7)
Duration [years]^†^	7.5 (3.2–11.8)	1.0 (0–3.5)	4.5 (0–9.0)	8.0 (1.1–14.9)	6.0 (2.5–8.5)	
*Disease types*						
Proctitis	6 (25.0%)			2 (50.0%)	2 (66.7%)	
Left-sided colitis	7 (29.2%)			0 (0%)	1 (33.3%)	
Right-sided colitis	0 (0%)			2 (50.0%)	0 (0%)	
Pancolitis	11 (45.8%)					
Ileitis		4 (26.7%)				
Ileocolitis		7 (46.7%)	4 (40%)			
Colitis		4 (26.7%)	6 (60%)			
*Part of biopsy*						
[*R*/*S*/*D*/*T*/*A/I*]	14/7/2/1/0/0	1/2/1/2/4/5	2/1/1/0/3/3	1/1/0/0/2/0	2/1/0/0/0/0	6/1/0/0/2/0
*Endoscopic index*						
REI	6.5 (5.0–8.0)			5.0 (3.6–6.4)	5.0 (2.5–7.5)	
SES-CD		10.0 (7.5–12.5)				
*Treatment*						
None	1	2	3	0	1	9
5-ASA	20	7	7	4	2	0
Corticosteroid	5	1	1	2	1	0
Thiopurine	1	0	1	0	1	0
Tacrolimus	1	0	0	0	0	0
Anti-TNFα	3	6	4	0	0	0

^†^ Median (interquartile range)

*R* rectum, *S* sigmoid colon, *D* descending colon, *T* transverse colon, *A* ascending colon, *I* ileum, *REI* *Rachmilewitz* endoscopic index, *SES-CD* simple endoscopic activity score for Crohn's disease

### Expression patterns of inflammatory mediators based on different intestinal locations

**Fig. 2:**
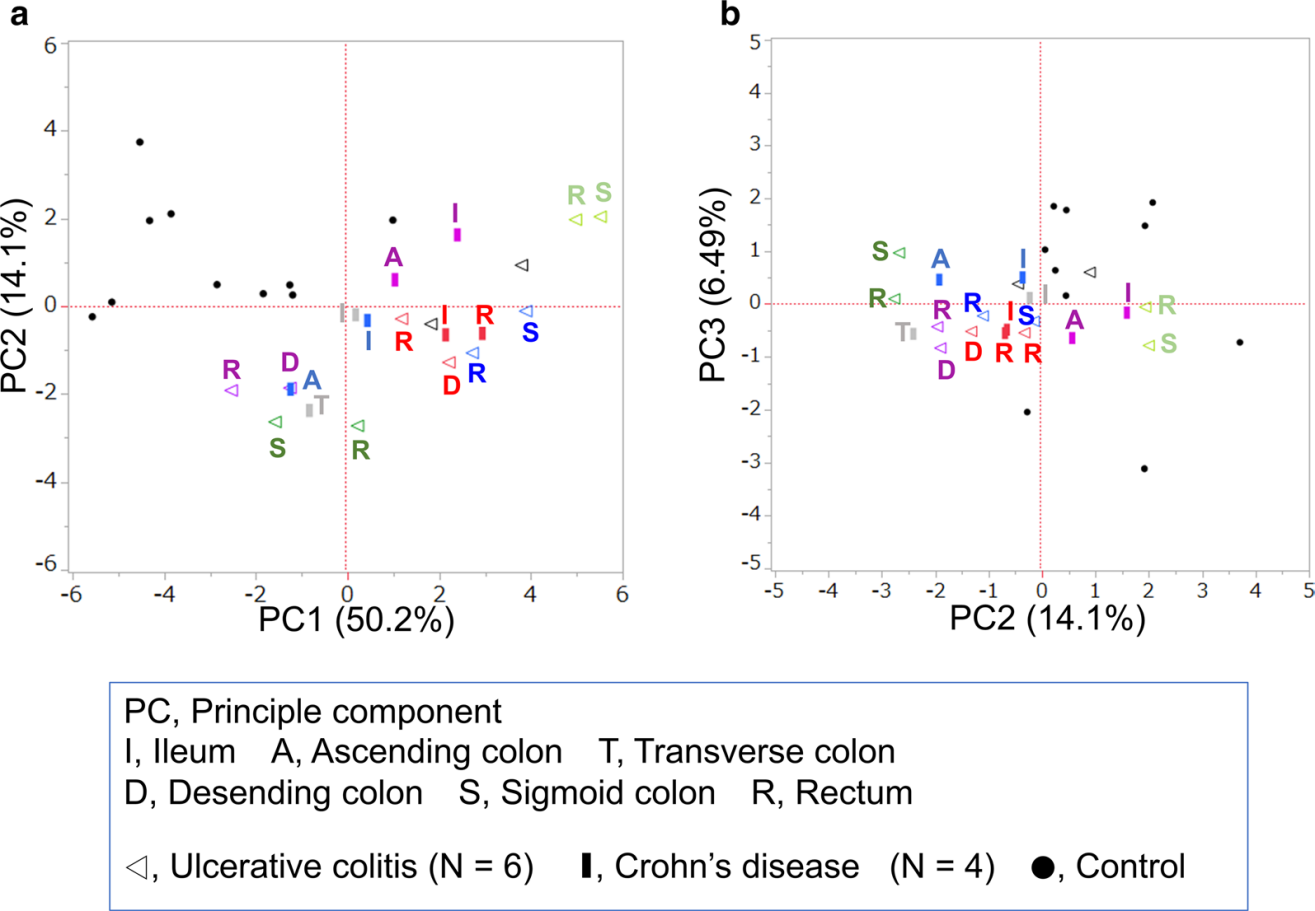
Expression patterns of inflammatory mediators in different locations of the intestine in the same patient. Selected PCs with discriminatory powers are shown as scatterplots, with 12 biopsy samples from 6 patients with UC, which are represented with triangles, and 8 biopsy samples from 4 patients with CD represented with squares. Same color indicates the same patient. A, Scatterplot of PC1 and PC2 obtained using 18 inflammatory mediators. B, Scatterplot of PC2 and PC3 obtained using 18 inflammatory mediators. Even in the colorectum and the ileum, where the mucosal background seems to be different, the expression of inflammatory mediators was found to be similar. *UC* ulcerative colitis, *CD* Crohn’s disease, *PC* principal component

We examined whether there was any difference in the expression patterns of inflammatory mediators based on the different locations, including the ileum, ascending colon, transverse colon, descending colon, sigmoid colon, and rectum in one patient each with UC or CD, where the ileum was evaluated exclusively in CD. PCA revealed that there are similar expression patterns of inflammatory mediators among them (Fig. 2). For CD, even between the colorectum and the ileum, there was no significant difference in the expression patterns of inflammatory mediators. From these results, all subsequent analyses were performed using one sample per patient.

### Evaluation of the differences in mRNA expression levels between UC and CD samples

Almost none of the inflammatory mediators showed significant differences the UC and the CD groups. However, the expression of IL-13 and IL-21 in the UC group was significantly higher than in the CD group (Additional file 1: Fig. S1). These results are consistent with those of our previous study [10], despite the subjects enrolled in a different period.

### Assessing the discriminant ability of IBD using multiple parameters of mucosally expressed inflammatory mediators

**Fig. 3:**
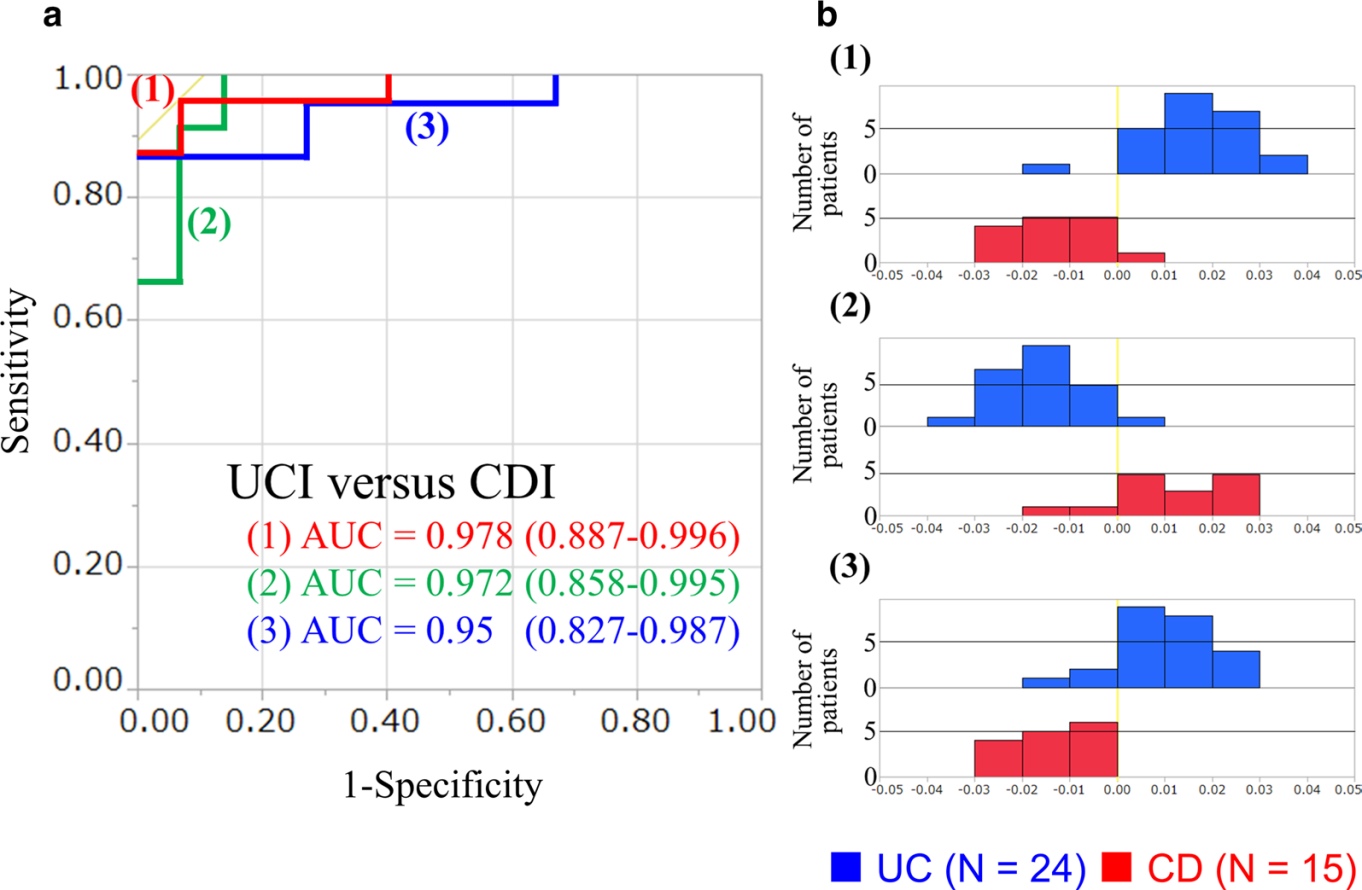
The ability of 3 multivariate markers to discriminate between UC and CD. A, ROC curves and AUC (95% CI) values of each multivariate marker set are shown. Discriminant Eq. (1) consisted of the 7 parameters was calculated as (− 2.1907*TNFa) + (0.0586*IL12p40) + (-2.0306*Tbet) + (0.32633*IL13) + (− 0.06193*GATA3) + (3.19025*IL21) + (0.42614*RORC) + 0.033857. Discriminant Eq. (2) consisted of 5 parameters was calculated as (1.70723*IFNg) + (1.100737*IL12p35) + (1.145*Tbet) + (0.42455*GATA3) + (− 3.9568*IL21) − 0.030844. Discriminant Eq. (3) consisted of 3 parameters was calculated as (-3.1947*Tbet) + (0.37669*IL13) + (2.06904*IL21) + 0.040244. B, Histograms showing the distribution of discriminant scores of each multivariate marker of UC and CD. Discriminant scores differentiated well between UC (blue) and CD (red). *ROC* receiver-operating characteristic curves

The seven-parameter discriminant equation (using TNF-α, IL-12 p40, T-bet, IL-13, GATA3, IL-21, and RORC), which were created using linear discriminant analysis (LDA) in our previous research [10], showed high accuracy in distinguishing between the UC and CD groups, achieving an AUC of 0.978. The seven- parameter discriminant equation enabled us to distinguish between UC and UD groups with a sensitivity of 95.8% and specificity of 93.3%. Similarly, the five-parameter discriminant equation (using IFN-γ, T-bet, IL-12 p35, GATA3, and IL-21) and the three-parameter discriminant equation (using T-bet, IL-13, and IL-21) also showed a high accuracy in distinguishing between the UC and CD groups, with AUC values of 0.972 and 0.95, respectively (Fig. 3). The five- and three-parameter discriminant equation enabled us to distinguish between UC and CD group with a sensitivity of 95.8% and 87.5%, and specificity of 86.7% and 100%, respectively.

In addition, 3D-scatterplots, analyzed using PCA, showed higher IL-13 and IL-21, and lower T-bet patterns in UC than the CD group (Additional file 1: Fig. S2), which was also consistent with our previous study [10]. From these results, it was considered that UC and CD can be distinguished only using reciprocal ΔCq values without using a calibrator sample.

### Ability of the three-parameter discriminant equation to diagnose IBD-U patients as UC or CD

It was found that the seven- and five-parameter discriminant equations diagnosed IBD-U patients as UC or CD correctly with a moderate probability of 60–70%, whereas the three-parameter discriminant equation achieved results with a high probability of 90% (Table 2).

**Table 2:** IBD-U patient identification results and accuracy rate

No	Final diagnosis	7 parameters	5 parameters	3 parameters
1	CD	Correct	Correct	Correct
2	UC	Correct	*Incorrect*	Correct
3	CD	Correct	Correct	Correct
4	CD	Correct	Correct	Correct
5	CD	*Incorrect*	Correct	Correct
6	UC	*Incorrect*	*Incorrect*	*Incorrect*
7	CD	Correct	*Incorrect*	Correct
8	CD	*Incorrect*	Correct	Correct
9	CD	*Incorrect*	Correct	Correct
10	UC	Correct	Correct	Correct
	Correct ratio	60%	70%	90%

### Discriminant analysis for PSC-UC and AIP-UC using mucosally expressed inflammatory mediators

**Fig. 4:**
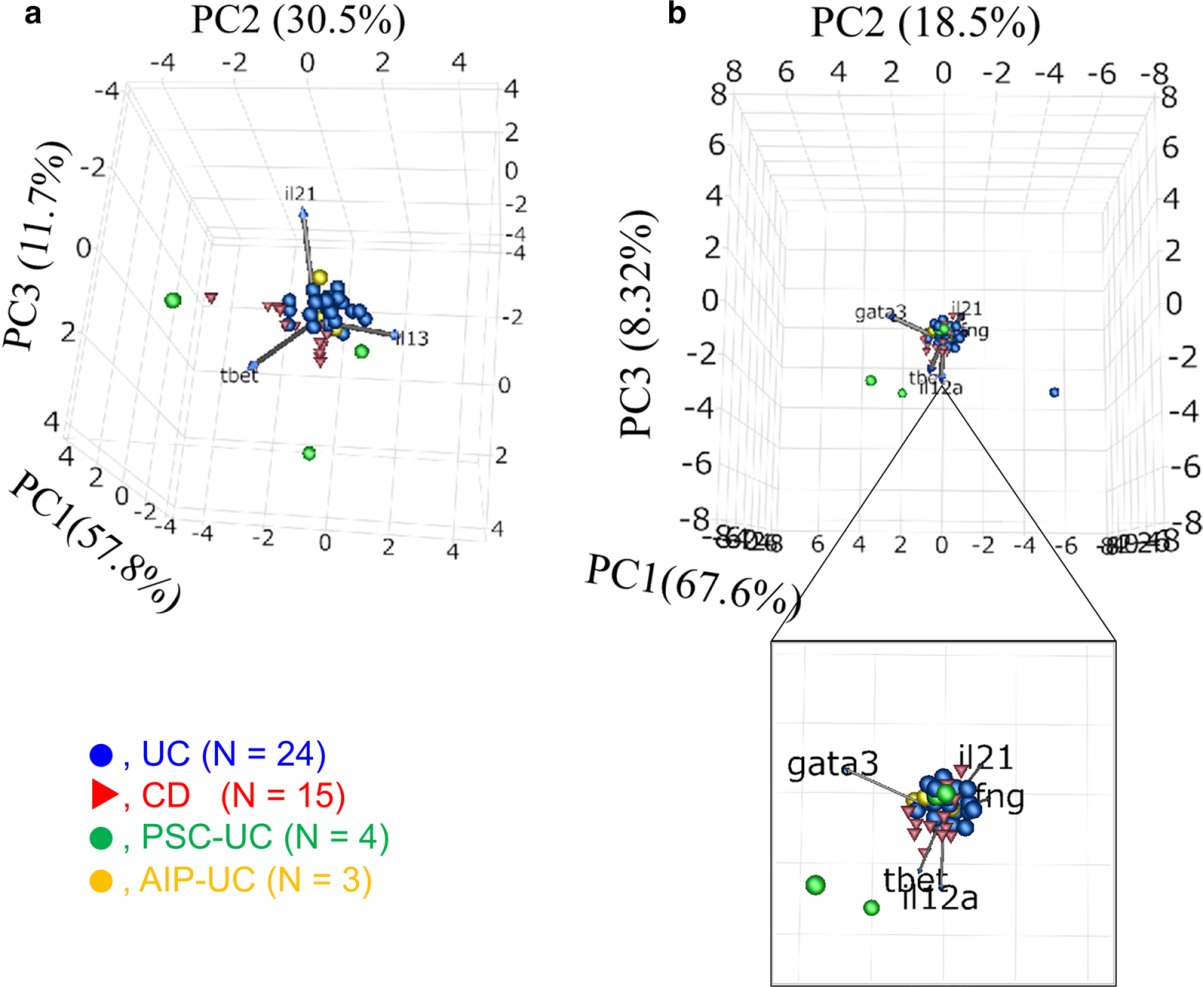
Visualization of differential expression patterns of the inflammatory mediators in the inflamed mucosa. Selected PCs with discriminatory abilities are shown as scatterplots; 24 patients with UC represented (blue dots), 15 patients with CD (red triangles), 4 patients with PSC-UC (green dots), and 3 patients with AIP-UC (yellow dots). A, 3D scatterplot of PCs obtained by the reconstruction of T-bet, IL-13, and IL-21. B, 3D scatterplot of PCs obtained by the reconstruction of T-bet, IL-12 p35, INFγ, IL-21, and GATA3. *UC* ulcerative colitis, *CD* Crohn’s disease, *PSC* primary sclerosing cholangitis, *AIP* autoimmune pancreatitis, *PC* principal component

Figure 4 shows 3D scatterplots of PCs with mucosally expressed inflammatory mediators along with the UC, CD, PSC-UC, and AIP-UC groups. The scatterplots of PSC-UC show lower IL-21 and higher T-bet than those of the UC, which are similar to those of CD. In contrast, the scatterplots of AIP-UC show higher IL-21 and lower T-bet than CD, which are similar to those of UC.

## Discussion

In the present study, we verified our previous results, where we described a model to discriminate between UC and CD [10]. We also showed that the three-parameter discriminant equation using T-bet, IL-13, and IL-21 had the potential to diagnose IBD-U as UC or CD with high accuracy, and assess the pathological conditions of IBD-like PSC-UC and AIP-UC. To our knowledge, this is the first study to create a diagnostic tool for IBD-U and the analysis of a specific type of IBD.

IBD-U is considered to be classified in several subtypes, depending on the clinical course and endoscopic findings. Those subtypes include a mixed phenotype between UC and CD, a UC-like phenotype with CD features, a CD-like phenotype with UC features, and transition phenotypes from UC to CD or CD to UC in the clinical course. However, it is difficult to classify IBD-U as UC or CD when the clinical findings are not typical for IBD. As the dysregulation of immune responses, resulting from the genetic background and environmental factors, plays a crucial role in the pathogenesis of IBD, it is reasonable that the analysis of the immune reactions enables us to make a differential diagnosis of IBD-U as UC or CD.

It is widely known that various Th-related genes are involved in IBDs [20–23], where UC and CD are a Th2/Th17 cells-related disease and a Th1/Th17 cell-related disease, respectively, according to the basic studies carried out using murine IBD models [24–27]. Furthermore, recent studies have revealed that Th17 cells can change their phenotype and repolarize towards a different condition, which is referred to as plasticity. For example, IL-12 and IL-23 can induce IFN-γ in IL-17-secreting Th17 cells, which are referred to as Th17/Th1 cells [28, 29]. The expressions of most Th-related genes in UC and CD overlap, and it was difficult to classify them via only the expression of specific genes.

We herein also showed that there is no significant difference in most inflammatory mediators between UC and CD (Additional file 1: Fig. S1). Even IL-13 and IL-21, which showed significant differences, could not differentiate UC from CD clearly by themselves [10]. Therefore, LDA was performed using seven, five, and three inflammatory mediators, and it was again verified that UC and CD can be distinguished with a high accuracy (Fig. 3). In addition, we created the discriminant equation using the reciprocal of ΔCq of each sample. Therefore, it should be noted that the discriminant equation that we created in this study could be a diagnostic tool that can be universally and directly used in clinical practice.

In this study, we analyzed the patients with IBD-U, who were eventually diagnosed with UC or CD after surgical operation, pathological results, and the clinical course of the disease. Notably, the seven- and five-parameter discriminant equations diagnosed patients with IBD-U as UC or CD correctly with a probability of 60–70%, whereas the three-parameter discriminant equation diagnosed patients correctly but with a high probability of 90%. The reason why the three-parameter discriminant equation showed higher probability than those of the seven- and five-parameter ones may be attributed to the diversity and heterogeneity of the population with IBD-U. Patients with IBD are considered a heterogeneous population; patients with IBD-U exhibit more heterozygous subtypes, such as CD-like UC and UC-like CD [1], although the characteristics of expression patterns of inflammatory genes in IBD-U has remain unclear. Therefore, IBD-U has phenotypic characteristics overlapping those of UC or CD may show genetic overlap in terms of inflammatory gene expression. In total, 5 items out of the seven-parameter discriminant Eq. (71.4%) and 4 items out of the five-parameter discriminant Eq. (80.0%) did not show significant differences between UC and CD (Additional file 1: Fig. S1). The greater the heterogeneous population is, such as IBD-U, the greater is the adverse effect of the proportion of parameters that are not significantly different between UC and CD. In contrast, in the three-parameter discriminant equation, only 1 out of the 3 parameters (33.3%) did not show significant differences between UC and CD (Additional file 1: Fig. S1, Fig. 3). Accordingly, the three-parameter discriminant equation, which had a high proportion of IL-13 and IL-21, was considered to be suitable for diagnosing IBD-U. Considering that bowel damage increases in patients with CD, it is important to apply an appropriate treatment as early as possible to prevent the progression of bowel damage [30]. Based on our findings, the three-parameter discriminant equation is useful for determining timely and appropriate treatment to patients with IBD-U long before a definitive diagnosis of CD is reached and can help avoid bowel damage progression.

In, addition to IBD-U, we also analyzed PSC-UC and AIP-UC which have unique pathological conditions of IBD. We visually analyzed the mucosal expression of inflammatory mediators in PSC-UC and AIP-UC using 3D scatterplots of PCs. As a result, the distribution graph of PSC-UC showing low IL-21 and high T-bet indicated that it was similar to conventional CD rather than conventional UC. This result is consistent with the findings reported previously [8, 9]. PSC-UC represents a different immunological disorder from UC, characterized by an increased intestinal Th1 response [8]. In contrast, the distribution graph of AIP-UC showing high IL-21 and low T-bet indicated that it was similar to conventional UC. These results indicate that UC may exhibit different inflammatory gene expressions depending on the complications and different subtypes. The visual distribution graph analysis may enable us to consider the treatment strategies for each subtype.

It was considered that mucosally expressed patterns of inflammatory genes in patients with IBD may differ depending on the intestinal portions including ileum, ascending colon, transverse colon, sigmoid colon and rectum. There are some differences in mucosal structure and environmental factors such as luminal contents and the microbiota. Surprisingly, however, the results of analysis for inflammatory genes in different intestinal portions of one patient have shown no significant differences in the mucosal expression patterns of inflammatory genes among the intestinal portion mentioned above (Fig. 2). Importantly, it indicates that any intestinal portion could be used for the discriminant analysis to make a differential diagnosis of IBD.

In addition, we investigated whether patient age was associated with the mucosal expression of inflammatory mediators. Correlation analysis revealed that age was not correlated with the mucosal expression of inflammatory mediators in UC (Additional file 1: Fig. S3A) or in CD (Additional file 1: Fig. S3B). Therefore, age does not affect the expression of inflammatory mediators. We also investigated whether anti-TNFα antibody treatment affected the mucosal expression of inflammatory mediators. Among the 49 patients with UC, CD, and IBD-U, 13 were treated with anti-TNFα antibody. No significant differences were observed in the mucosal expression of inflammatory mediators between patients subjected to anti-TNFα antibody treatment and those not subjected to anti-TNFα antibody treatment (Additional file 1: Fig. S4). There are several limitations to the present study. First, this was a retrospective study. Second, to obtain a more appropriate discriminant coefficient, it is necessary to use a larger number of patients. Furthermore, we herein analyzed only 18 genes. Finally, the relatively small number of patients with IBD-U enrolled in this study may have introduced bias. Based on our results, a large-scale prospective study is required to confirm that the three-parameter discriminant equations are useful in clinical practice.

## Conclusion

The discriminant equation using mucosally expressed cytokines of IL-13, IL-21, and T-bet created in this study can be used as a universal diagnostic tool not only to make a differential diagnosis of IBD, UC or CD for IBD-U but also to assess pathological conditions in PSC-UC and AIP-UC. We propose that the discriminant analysis has a potential for becoming an objective universal diagnostic tool for subtypes of IBD in future clinical practice.

## Supplementary Information

**Additional file 1: Table S1**. List of genes analyzed in biopsy samples by quantitative real-time PCR. **Table S2**. Clinical course of IBD-U patients. **Fig. S1**. Mucosally expressed 18 T cell-related mRNAs in active UC, active CD, and non-colitis control groups. **Fig. S2**. Visualization of differential expression patterns in the inflamed mucosa via principle component analysis. **Fig. S3**. Correlations between age and inflammatory gene expression in patients with IBD. **Fig. S4**. Comparison of 18 mucosally-expressed T cell-related mRNAs with and without anti-TNFα antibody.

## Data Availability

The datasets used and/or analysed during the current study are available from the corresponding author on reasonable request.

## Abbreviations

IBD: Inflammatory bowel diseases
UC: Ulcerative colitis
CD: Crohn’s disease
IBD-U: Inflammatory bowel disease-unclassified
PSC-UC: UC along with primary sclerosing cholangitis
AIP-UC: UC along with autoimmune pancreatitis
REI: Rachmilewitz endoscopic index
SES-CD: Simple endoscopic score for CD
Cq: Threshold cycle
PCA: Principal component analysis
PCs: Principal components
LDA: Linear discriminant analysis
AUCs: Areas under the receiver operating characteristic curves
