# Supplementary material for: Discriminant equation using mucosally expressed cytokines and transcription factor for making definite diagnosis of inflammatory bowel disease unclassified
Source: BMC Gastroenterol. 2021 Feb 16;21:73. doi: 10.1186/s12876-021-01656-1 (PMC7885231; doi:10.1186/s12876-021-01656-1)
Supplement: Supplementary file 1 — Additional file 1: Table S1. List of genes analyzed in biopsy samples by quantitative real-time PCR. Table S2. Clinical course of IBD-U patients. Fig. S1. Mucosally expressed 18 T cell-related mRNAs in active UC, active CD, and non-colitis control groups. Fig. S2. Visualization of differential expression patterns in the inflamed mucosa via principle component analysis. Fig. S3. Correlations between age and inflammatory gene expression in patients with IBD. Fig. S4. Comparison of 18 mucosally-expressed T cell-related mRNAs with and without anti-TNFα antibody. [file 12876_2021_1656_MOESM1_ESM.docx]

**Discriminant equation using mucosally expressed cytokines and transcription factor for making definite diagnosis of inflammatory bowel disease unclassified**

Hiroaki Okuno ^a,b^ Haruei Ogino ^a^ Eikichi Ihara ^a,c^ Kei Nishioka ^a^ Yoshimasa Tanaka ^a^ Takatoshi Chinen ^a^ Motoyuki Kohjima ^a^ Takamasa Oono ^a^ Masatake Tanaka ^a^ Takeshi Goya ^a^ Nao Fujimori ^a^ Yoichiro Iboshi ^d^ Takuji Gotoda ^b^ Yoshihiro Ogawa ^a^

^a^ Department of Medicine and Bioregulatory Science, Graduate School of Medical Sciences, Kyushu University, Fukuoka, Japan.

^b^ Division of Gastroenterology and Hepatology, Department of Medicine, Nihon University School of Medicine, Tokyo, Japan.

^c^ Department of Gastroenterology and Metabolism, Graduate School of Medical Sciences, Kyushu University, Fukuoka, Japan.

^d^ Department of Gastroenterology, Clinical Research Institute, National Hospital Organization, Kyushu Medical Center, Fukuoka, Japan.

Correspondence: Haruei Ogino, MD, PhD, Department of Medicine and Bioregulatory Science, Graduate School of Medical Sciences, Kyushu University, 3-1-1 Maidashi, Higashi-ku, Fukuoka 812-8582, Japan.

E-mail: [ogino@intmed3.med.kyushu-u.ac.jp](mailto:ogino@intmed3.med.kyushu-u.ac.jp)

Tel: +81-92-642-5278, Fax number: +81-92-642-5287

**Supplementary online contents**

**Additional file 1: Table S1.** List of genes analyzed in biopsy samples by quantitative real-time PCR.

**Additional file 1: Table S2.** Clinical course of IBD-U patients.

**Additional file 1: Fig. S1.** Mucosally expressed 18 T cell-related mRNAs in active UC, active CD, and non-colitis control groups.

**Additional file 1: Fig. S2.** Visualization of differential expression patterns in the inflamed mucosa via principle component analysis.

**Additional file 1: Fig. S3.** Correlations between age and inflammatory gene expression in patients with IBD.

**Additional file 1: Fig. S4.** Comparison of 18 mucosally-expressed T cell-related mRNAs with and without anti-TNFα antibody.

| **Additional file 1: Table S1.** List of genes analyzed in biopsy samples by quantitative real-time PCR. | | |
| --- | --- | --- |
|  | Genes | ABI Assay Number^†^ |
| Th1 cytokine genes | *TNFA* (TNF-α) | Hs00174128_m1 |
|  | *IFNG* (IFN-γ) | Hs00989291_m1 |
|  | *IL12A* (IL-12 p35) | Hs00168405_m1 |
|  | *IL12B* (IL-12 p40) | Hs01011518_m1 |
| Transcription factor | *TBX21* (T-bet) | Hs00203436_m1 |
| Th2 cytokine genes | *IL5* (IL-5) | Hs01548712_g1 |
|  | *IL13* (IL-13) | Hs00174379_m1 |
|  | *IL33* (IL-33) | Hs01125943_m1 |
| Transcription factor | *GATA3* (GATA3) | Hs00231122_m1 |
| Th17 cytokine genes | *IL17A* (IL-17A) | Hs00174383_m1 |
|  | *IL17F* (IL-17F) | Hs00369400_m1 |
|  | *IL21* (IL-21) | Hs00222327_m1 |
|  | *IL22* (IL-22) | Hs01574154_m1 |
|  | *IL23A* (IL-23 p19) | Hs00413259_m1 |
|  | *IL6* (IL-6) | Hs00174131_m1 |
| Transcription factor | *RORC* (RORC) | Hs00172860_m1 |
| Treg cytokine genes | *TGFB* (TGF-β) | Hs00171257_m1 |
| Transcription factor | *FOXP3* (FoxP3) | Hs00203958_m1 |
| Endogenous control | *18S* (ribosomal RNA) | Hs99999901_s1 |
| Gene symbols are italicized.  ^†^Applied Biosystems gene expression assay number.  IFN, interferon; FoxP, forkhead box P. | | |

| **Additional file 1 Table S2.** Clinical course of IBD-U patients. | | | |  |  |  |  |  |
| --- | --- | --- | --- | --- | --- | --- | --- | --- |
| No | Sex | Age | Clinical Course | | Initial Diagnosis | Final  Diagnosis | Duration for  Final Diagnosis |  |
| 1 | F | 36 | At first, there was no ileal lesion, but a longitudinal ulcer and continuous inflammation were found only in the colon. A biopsy was performed. During the course, ulcerative lesions of the ileum and cobblestone appearance became prominent. | | CD on UC suspect | CD  Ileo-colitis | 3 years |  |
| 2 | M | 44 | At first, there was local diffuse inflammation and a slightly longitudinal ulcer only in the colon. He also had an anal fistula. A biopsy was performed. Surgical intervention was performed owing to long-term inflammation. There was inflammation with loss of goblet cells, but no granulomas in postoperative specimens. | | CD on UC suspect | UC  Colitis | 11 years |  |
| 3 | M | 15 | After perianal abscess treatment, he was examined at several hospitals. There was only diffuse inflammation in sigmoid colon, which did not meet the diagnostic criteria for IBD. A biopsy was performed. After TCS in our hospital, a longitudinal ulcer and epithelioid cell granulomas were observed. | | IBD-U | CD  Colitis | 4 years |  |
| 4 | F | 26 | Had persistent diarrhea and abdominal pain, was being examined at another hospital. There was sever colon inflammatory, which did not meet the diagnostic criteria for IBD. A biopsy was performed. Further examination revealed a longitudinal ulcer and cobblestone appearance of the colon and week inflammation of the ileum. | | UC on CD suspect | CD  Ileo-colitis | 6 years |  |
| 5 | M | 50 | At first, there was no ileal lesion and histological features of IBD, but a local cobblestone appearance was found in the ascending colon. The potential of infection, ischemic colitis and colon cancer were excluded. A biopsy was performed. During the course, discontinuous cobblestone appearance was seen. | | IBD-U | CD  Colitis | 7 years |  |
| 6 | F | 30 | At first, there was a longitudinal ulcer in the colon and the patients was diagnosed with CD at another hospital. A biopsy was performed. During the course, continuous lesions of the colon became prominent, but no lesion appeared in the ileum. | | CD suspect | UC  Colitis | 8 years |  |
| 7 | M | 29 | At first, week continuous diffuse erosion and ulcers were observed in the ileum, while this was stronger in the colon. A biopsy was performed. During the course, ileal lesions became prominent and colon lesion seems skip pattern. | | IBD-U | CD  Ileo-colitis | 2 years |  |
| 8 | M | 39 | At first, there was a longitudinal ulcer and diffuse erosion from the sigmoid to ascending colon. A biopsy was performed. During the course, a longitudinal ulcer appeared in a skip pattern and became prominent, which was not considered as continuous lesion. | | IBD-U | CD  Colitis | 2 years |  |
| 9 | M | 37 | At first, there was aphthae in the terminal ileum and diffuse inflammation in the cecum. Therefore, he did not meet diagnostic criteria of IBD at another hospital. A biopsy was performed. A few years later, surgical intervention was performed owing to stenosis, ulcer, and gastrointestinal perforation. | | IBD-U | CD Ileo-colitis | 14 years |  |
| 10 | M | 36 | Diarrhea persisted from a young age and he was diagnosed with IBS at multiple medical institutions. TCS revealed diffuse erosion only in the transverse colon and ileum. A biopsy was performed. During the course, continuous lesions of the colon became prominent, while ileal lesion had disappeared. | | IBD-U | UC  Colitis | 12 years |  |
| IBD-U, Inflammatory bowel disease unclassified; UC, ulcerative colitis; CD, Crohn’s disease; TCS, Total colonoscopy; IBS, irritable bowel syndrome. | | | | | | | |  |


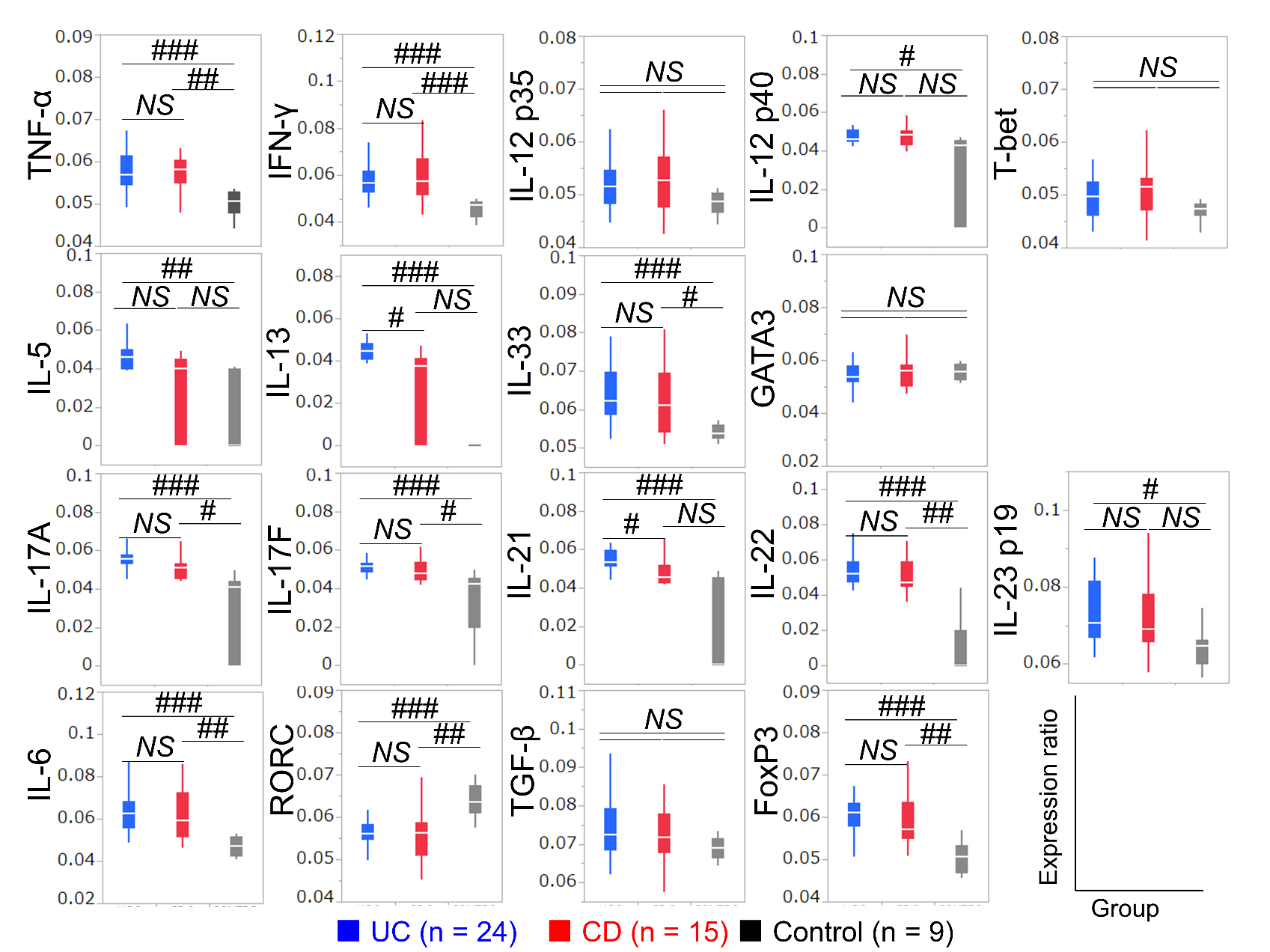


**Additional file 1: Fig. S1.** Mucosally expressed 18 T cell-related mRNAs in active UC, active CD, and non-colitis control groups.

Blue bar indicates UC, red bar indicates CD, and the black bar indicates control group.

The expression is shown as “reciprocal ΔCq,” where the difference of 1 in the value shows a 2-fold difference in the expression level. When expression was not detected, a value of 0 was given. The UC and CD groups show significant high expression of many targets compared to the control group. Only IL-13 and IL-21 have a higher expression in UC than in CD.

UC, ulcerative colitis; CD, Crohn’s disease; ###, P <0.001; ##, P <0.01; #, P <0.05; *NS*, not significant.


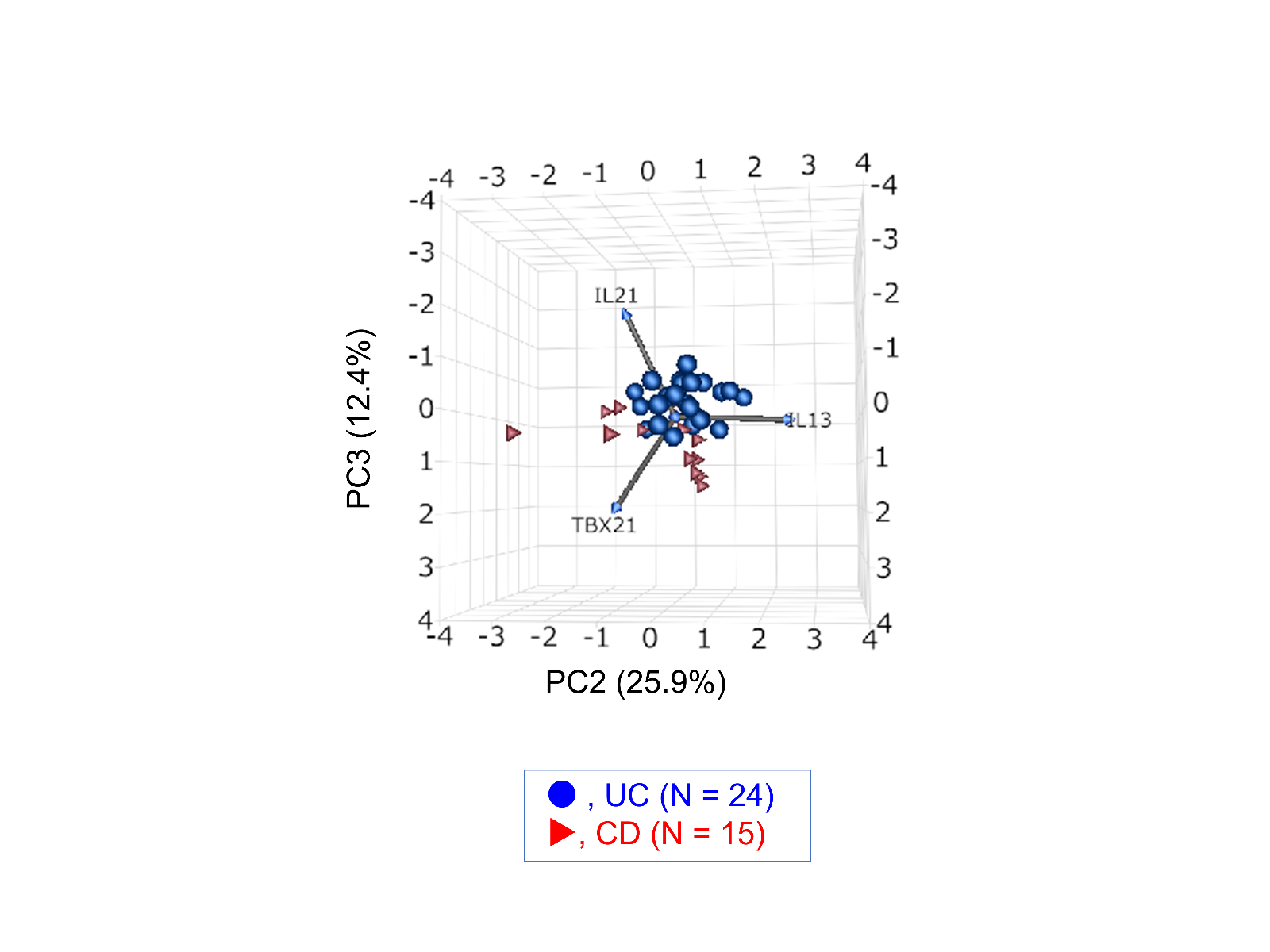


**Additional file 1: Fig. S2.** Visualization of differential expression patterns in the inflamed mucosa via principle component analysis.

Selected PCs with discriminatory powers are shown as scatterplots, with 24 UC patients represented as blue dots and 15 CD patients as red triangles. A, Scatterplot of PC2 and PC3 obtained by reconstruction of T-bet, IL-13, and IL-21.

UC, ulcerative colitis; CD, Crohn’s disease; PC, principal component.


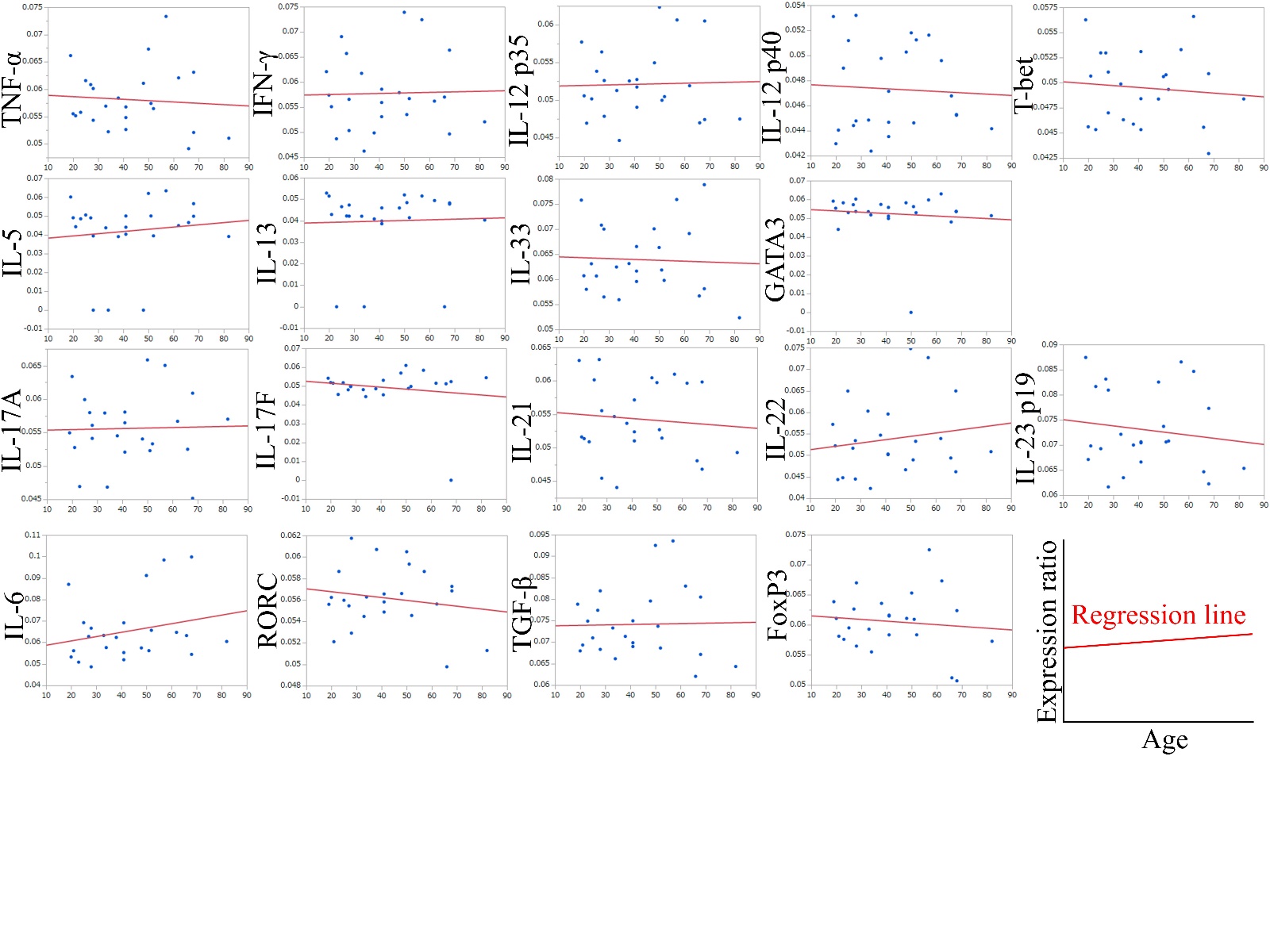
A.


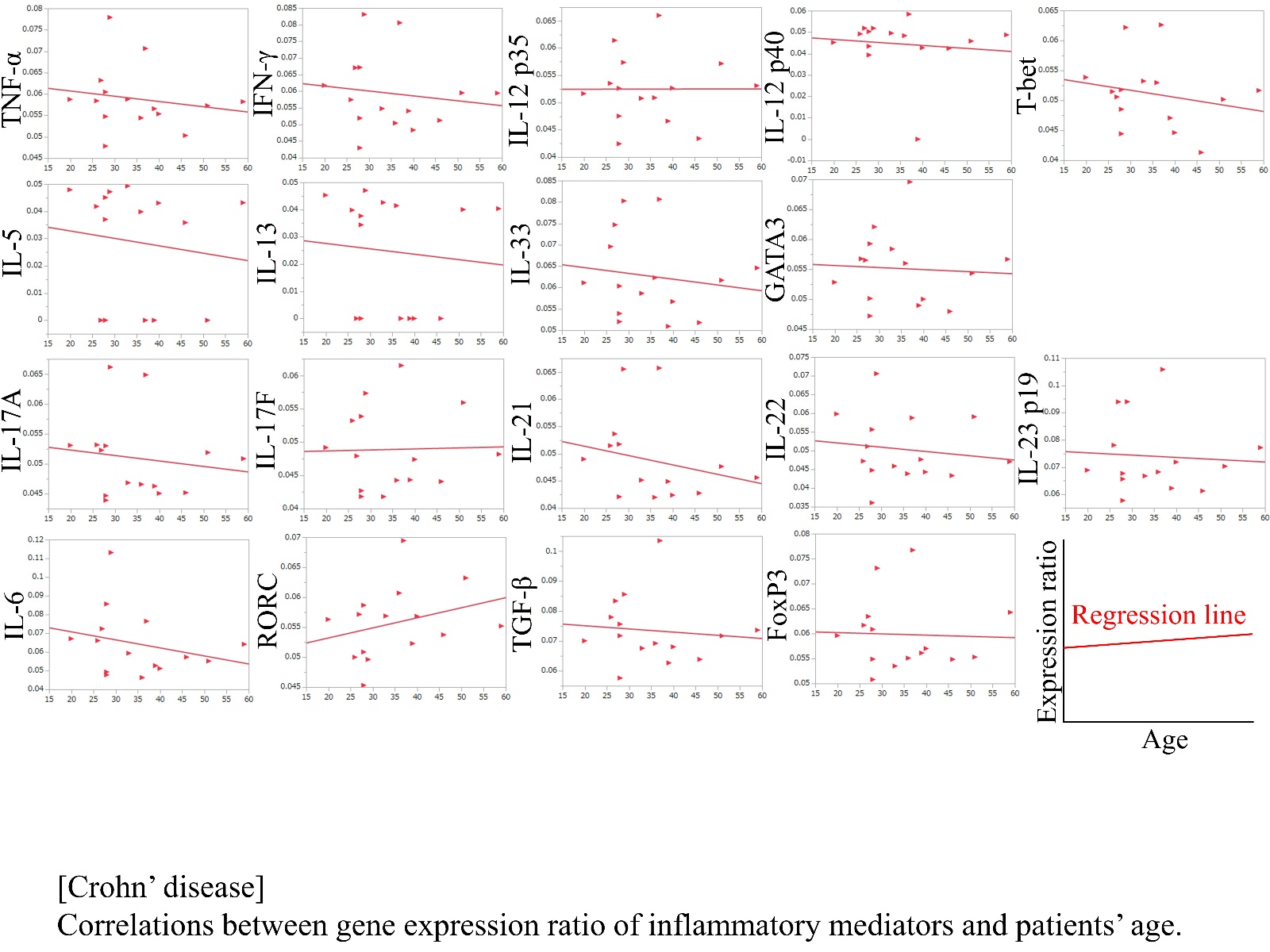
B.

**Additional file 1: Fig. S3.** Correlations between age and inflammatory gene expression in patients with IBD.

A, B, Correlations between age and inflammatory gene expression in patients with ulcerative colitis (A) and Crohn’s disease (B) are shown.

R^2^, coefficient of determination.


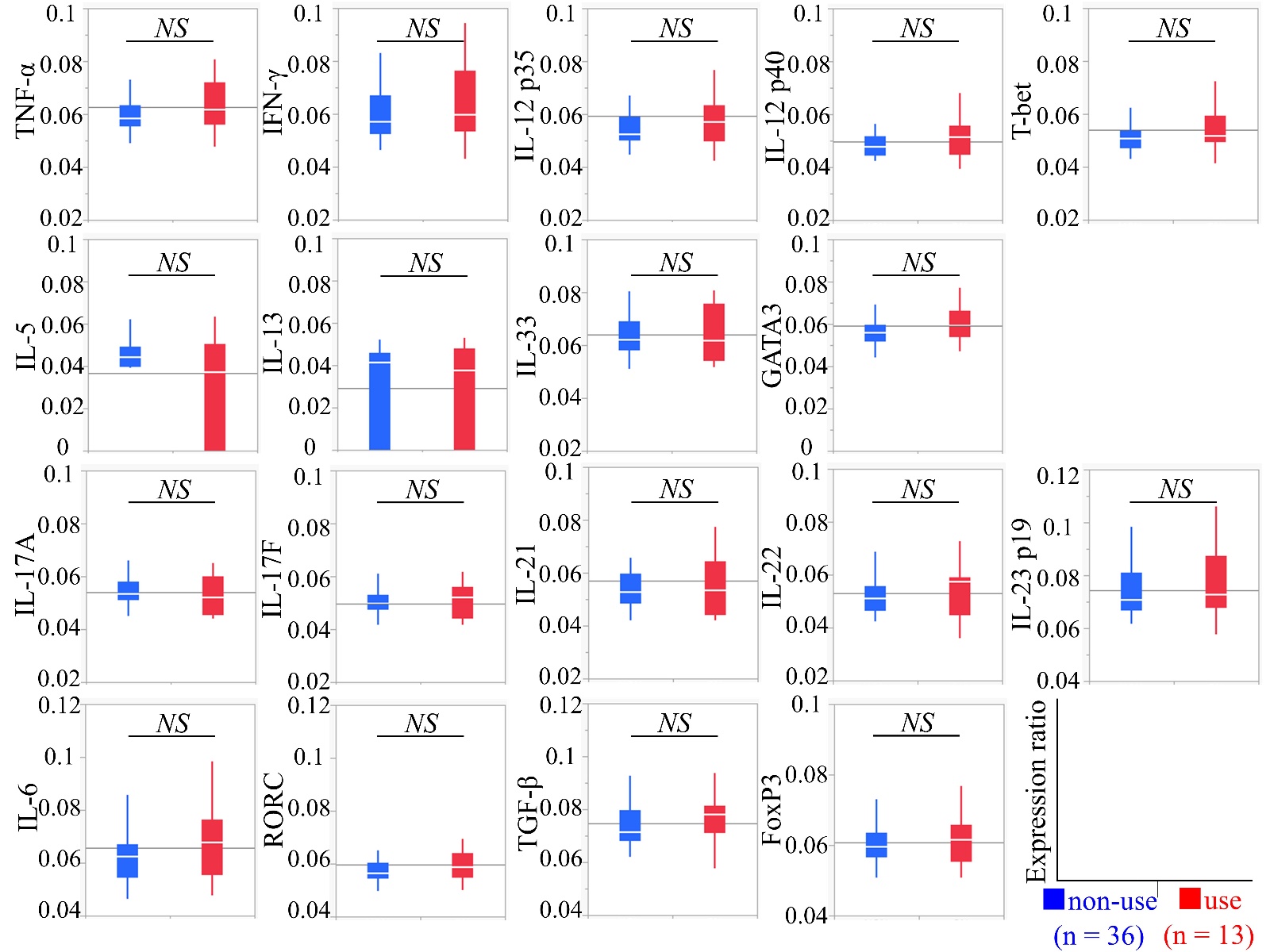


**Additional file 1: Fig. S4.** Comparison of 18 mucosally expressed T cell-related mRNAs in patients subjected to and those not subjected to anti-TNFα antibody treatment.

Expression of indicated mucosally expressed T cell-related mRNAs in patients not treated with (blue bar) and those treated with (red bar) anti-TNFα antibody. The expression level is shown as “reciprocal ΔCq”, where a difference of 1 indicates a 2-fold difference in the expression level.

NS, not significant.
